# Determinants of enrollment of informal sector workers in cooperative based health scheme in Bangladesh

**DOI:** 10.1371/journal.pone.0181706

**Published:** 2017-07-27

**Authors:** Abdur Razzaque Sarker, Marufa Sultana, Rashidul Alam Mahumud, Sayem Ahmed, Ziaul Islam, Alec Morton, Jahangir A. M. Khan

**Affiliations:** 1 Health Economics and Financing Research, International Centre for Diarrhoeal Disease Research, Bangladesh (ICDDR,B), Dhaka, Bangladesh; 2 University of Strathclyde, Glasgow, Scotland; 3 Karolinska Institute, Stockholm, Sweden; 4 Liverpool School of Tropical Medicine, Liverpool, United Kingdom; Imperial College London, UNITED KINGDOM

## Abstract

**Background:**

Providing access to affordable health care for the informal sector remains a considerable challenge for low income countries striving to make progress towards universal health coverage. The objective of the study is to identify the factors shaping the decision to enroll in a cooperative based health scheme for informal workers in Bangladesh and also help to identify the features of informal workers without health schemes and their likelihood of being insured.

**Methods:**

Data were derived from a cross-sectional in-house survey within the catchment area of a cooperative based health scheme in Bangladesh during April–June 2014, covering a total of 784 households (458 members and 326 non-members). Multivariate logistic regression model was used to identify factors associated with cooperative based health scheme and explanatory variables.

**Findings:**

This study found that a number of factors were significant determinants of health scheme participation including sex of household head, household composition, occupational category as well as involvement social financial safety net programs.

**Conclusion:**

Findings from this study can be suggestive for policy-makers interested in scaling up health insurance for informal workers in Bangladesh. Shared funding from this large informal sector can generate new resources for healthcare, which is in line with the healthcare financing strategy of Bangladesh as well as the recommendation of the World Health Organization for developing social health insurance as part of the path to Universal Health Coverage.

## Background

Effectiveness of healthcare delivery and overall health system performance are closely tied to healthcare financing mechanisms [1]. Healthcare financing schemes include direct payment of households and social, private and community based health insurance through which health services are obtained by households. In Bangladesh, out of pocket health expenditure by households (OOP) constitutes the major component of total health spending and the OOP share of total health expenditure has increased from 55.9% in 1997 to 59.9% in 2005 to 63.3% in 2012 according to the latest national health accounts survey [2]. Reliance on out-of-pocket expenditure for health services leads to a catastrophic burden for many households in Asia including Bangladesh [3]. There is growing international consensus on the importance of social protection in health to the whole population in order to minimize financial barriers to healthcare for needy and to avoid catastrophic health expenditures [4–7]. Government revenue through tax and micro health insurance are considered as two possible mechanisms for financing healthcare of low income people. However, inclusion of low income people (especially informal sector workers) in the tax system is still a challenge as they are not well organized, geographically dispersed, not part of any one organization and even not properly tracked in formal way through systems of national registration. Further, it is not easy to assess their income properly, on the basis of which social contributions can be deducted [8]. Providing access to affordable health care for the informal sector remains a considerable challenge for low income countries striving to make progress towards universal health coverage. Considering the contribution of these workers to the economy of Bangladesh, where informal sector workers (in agriculture and non-agriculture sector) alone constitute 88% of total labor force and represent industries worth 64% of GDP, it is important to make an effort to attract these people towards a self-financed health scheme. The Healthcare Financing Strategy of Bangladesh proposed population coverage in conjunction with financing mechanisms for the country, showing that 85.7 Million people (56.2% of total population) or 19.0 Million households are connected with informal sector and may be able to afford small premium payments for existing or new community-based health, micro health insurance or other innovative initiatives as a means to purchase financial protection against catastrophic expenditures that cause impoverishment. In recent years, community based health insurance has been promoted within health financing reforms in many developing countries including Bangladesh. In fact, the concept of community based health schemes has attracted increasing attention from policy makers in the recent past. Such schemes are now recognized as initiatives that can be both community friendly and have a wider reach than other health insurance schemes in the informal sector especially if well designed [9]. Occupational associations can play an important role in engaging such workers for healthcare financing [10,11]. Specifically, the cooperative model is well-suited for developing community-based schemes and reaching a large number of informal sector workers for developing community-based health financing schemes [12,13]. Community based Health Insurance can be thought of as a risk management strategy, that works on the basic principle of pooling of risks of unexpected costs of persons falling ill and needing hospitalization by charging a premium from a wider population base of the same community [14,15]. Despite the proven effects of community based health insurance scheme in enhancing to service utilization, financial protection, quality of care and even cost recovery, securing health enrollment in such schemes is critical and represents a challenge in developing countries [16–19].The determinants of low participation of individuals in the informal sector is attributed to a number of factors, including low and non-regular incomes, insecure employment, as well as, education, household wealth status, marital status, age, exposure to the mass media, household head, place of residence and also insurance scheme design features such as inflexible payment schedules and lack of awareness [8,20–23]. Furthermore, perception of the adequacy of traditional care (e.g. traditional healers and traditional medicine), distance from the health facility, and level of socioeconomic inequality within the community also impacts on the enrollment in such health insurance schemes [24]. Although much literature mentions determinants of health insurance of informal sector in low- and middle-income countries (LMICs), the determinants associated with health insurance ownership among informal sector workers in Bangladesh has received little attention. The aim of this study is to identify factors shaping the decision to enroll in a cooperative based health scheme for informal workers in Bangladesh and can also help to identify the features of informal workers without health schemes and their likelihood of being insured.

## Methods

### Study setting

The study was conducted in Chandpur district of Bangladesh where the implementation of a cooperative based health scheme (Co-BHS) is ongoing. Workers with informal employment (including self-employment) are targeted by the scheme. Encouraged by an earlier project educating informal sector workers about health insurance mechanism, its utility and usage of the cooperative model [12], a group of workers established a health cooperative in Chandpur sub-district, named *Labor Association for Social Protection* (LASP). Membership of LASP is open for informal workers who pay a membership fee. The subscription unit for insurance is the household rather than the individual. One member of a household can pay a membership fee weekly, monthly or quarterly and if they missed a payment in this period, they can repay it along with the next payment scheduled. The fee (per household) is fixed based on the willingness-to-pay of informal workers for health insurance and a minimum of ten Bangladeshi Taka (0.128 USD) per household per week is charged. For one membership, up to 6 members of the household are considered as beneficiaries. However, additional household members can be enrolled by paying extra premium (BDT 2) per person per week. There is a uniform benefit package for all member of LASP and a waiting period of the two weeks after a first payment before making a claim. Access to qualified medical doctors (MBBS), medicine, diagnostic tests and inpatient care are included in the benefit package at a subsidized price and uniform financial support, i.e. maximum BDT 4,000 (51.29 USD) is provided per household per year for inpatient care. In addition to the cooperatives’ own doctors and pharmacy, private and public healthcare providers are contracted for services to the members. A group of specialized doctors such as pediatric specialists, gynecology specialists, cardiologists, endocrinologists and more are also contracted.

### Study design and sampling

A cross-sectional in-house survey was conducted within the catchment area of LASP under 14 unions (the lower administrative level in rural areas) and a municipality in Chandpur sub-district of Bangladesh during April–June 2014, covering a total of 784 households (458 members of CBHI and 326 non-insured households). We sought non-insured households which were matched with the insured households in terms of residence (same village/union), occupational attachment, income level, demographic structure and household composition (under-five children, presence of female members at reproductive age and elderly (60 years and above) person. To ensure the best possible matching, first two characteristics (residence and occupation) were a precondition for matching and then the demographic and household composition was considered.

### Study population

The study population have been selected based on two major criteria- first, the household with incomes falling in the lowest taxable income bracket and secondly, with at least one blue-collar informal worker who also meets for inclusion criteria for being a member of this cooperative. The determinants were measured among household head, who was determined by the particular household aged 18 and older. Since household had been the functional unit for income and expense, data was collected and analyzed at the household level [25].

### Data collection and variables

All data collection tools were pretested for quality control and field interviewers and supervisors were trained and supervised by two field investigators. Two categories of data were collected through a structured questionnaire. The first category was socio-economic demographics of household and personal characteristics of household head, who represent important economic contributors as well as the decision makers of the household. The outcome variable was whether a household was covered by cooperative based health scheme (yes or no). The explanatory variables examined in this study were selected based on factors cited in the similar literature as influencing the decisions to participate this scheme including sex, age, education, marital status, occupation, household size, household wealth status, community as well as social financial safety net programs (i.e. receipt of governmental financial support due to age, veteran status, widowhood or the like). Age was considered in five groups, in particular; 18–25 years, 26–35 years, 36–45 years, 46–64 years or 65 and more years. Marital status was categorized into two groups; married or other (unmarried, widowed, divorced or separated). Educational level was captured as no education, primary education, secondary education, higher secondary or higher grade education. Occupation status was classified into six groups; formal employment, worker, business, house wife, farmer or other (unemployment, students, etc).

The second category of data was on quality of life related data on individual level to capture the health related quality of life (HRQoL) values using the EQ-5D instruments. Similar instrument was used in earlier studies to evaluate the health insurance intervention [26,27]. The EQ-5D is a generic health related quality of life HRQoL instrument which was applied for assessing self-reported health status using the five dimensions of self-reported health such as mobility, self-care, ability to perform usual activities, pain or discomfort and anxiety or depression [28]. The responses to each dimension were classified into three levels of severity– 1 indicates “no problem,” 2 indicates “some problem” and 3 indicates “extreme problem.” The developers of the EQ-5D have generated value sets in several countries to calculate a preference-based index for the 243 health states defined by responses to the 5 questions of the EQ-5D, where values of EQ-5D, health weight ranges between -0.594 (worst possible score) and 1 (best possible score) representing overall quality of life and higher scores in EQ-5D mean better quality of life, however, negative score indicates that sometimes the health condition is worse than death. For entire analyses, EQ-5D weight were grouped into three categories: less than 0.50, 0.50 to 0.89 and 0.90 to 1.00 respectively. The minimal dataset underlying the findings of this study has been provided as supporting information (see S1 dataset).

### Statistical analysis

Descriptive statistics were computed to summarize the data in relation to the different variables.

For explanatory variables, the category found to be at lower risk in the odds of the having a enrollment status of health scheme was selected as the reference group. Logistic regression was performed using all factors associated with enrollment status in an initial unadjusted and adjusted analysis after considering potential confounders. Goodness of fit was assessed using the Hosmer and Lemeshow statistic [29]. Both unadjusted odds ratio (for univariate analysis) and adjusted odds ratio (for multivariate analysis) with 95% confidence interval (CI) were calculated to measure associations. Variance Inflation Factor (VIF) test was done to determine whether multicollinearity was present or not. In statistical analysis for choosing matching variables, we have performed some exploratory analyses on the distribution of the variable [30]. Data cleaning, validation and all statistical analysis were performed using the STATA 13.0 software.

### Ethical approval

The research protocol of this study was approved by the Institutional Review Board of the International Centre for Diarrhoeal Disease Research, Bangladesh (icddr,b). All respondents were given an information sheet in Bengali explaining their rights relating to their voluntary participation in the study and were asked to sign a consent form.

## Results

### Characteristics of the study population

Table 1 demonstrates the result of the background characteristics of the study population. A total sampled population of 784 household heads including male (75.77%) and female (24.23%) were analyzed in this study. Among the total respondents (N = 784), about 75.77% were male and 24.23% were female-headed households. Higher proportions of the respondents (33.29%) were aged between 26 to 35 years and 43.49% had a primary level of education followed by 36.10% had a secondary level. Most of the respondents were married (88.65%) and most households had 5–6 members (54.59%). Among the total respondents, workers (e.g. rickshaw-puller, hotel workers, van drivers) accounted for 32.53% and the rests were self-employed, housewives, farmers, informal employment (below the tax threshold) and other. Nearly half of the respondents (49.62%) had reported their health weight ranges between 0.5 to .89 followed by the better health ranges 0.9 and above (39.41%). More than 58% of respondents lived in rural communities. According to income quintile of their earnings level 24.87% were in the poorest and 18.88% were in the richest income groups among the respondent.

**Table 1:** Characteristics of Co-BHS members and non-member households.

Variables	n (%)	95% CI
**Sex**		
*Male*	594 (75.77)	(72.63, 78.64)
*Female*	190 (24.23)	(21.36, 27.37)
**Age**		
*18–25*	100 (12.76)	(10.59, 15.28)
*26–35*	261 (33.29)	(30.07, 36.67)
*36–45*	196 (25.00)	(22.09, 28.16)
*46–64*	193 (24.62)	(21.72, 27.76)
*≥65*	34 (4.34)	(3.11, 6.01)
**Marital status**		
* Married*	695 (88.65)	(86.23, 90.69)
* Other*	89 (11.35)	(9.31, 13.77)
**Highest education level**		
* No education*	97 (12.37)	(10.24, 14.87)
* Primary*	341 (43.49)	(40.05, 47.00)
* Secondary*	283 (36.10)	(32.8, 39.53)
* Higher secondary*	36 (4.59)	(3.33, 6.30)
* Higher*	27 (3.44)	(2.37, 4.98)
**Household size**		
* ≤ 2*	23 (2.93)	(1.96, 4.38)
* 3–4*	223 (28.44)	(25.39, 31.71)
* 5–6*	428 (54.59)	(51.08, 58.06)
* 7–8*	88 (11.22)	(9.19, 13.64)
* > 8*	22 (2.81)	(1.85, 4.23)
**Occupational status**		
*Worker*	255 (32.53)	(29.33, 35.89)
*Business*	159 (20.28)	(17.61, 23.25)
*House wife*	156 (19.90)	(17.24, 22.85)
*Farmer*	57 (7.27)	(5.65, 9.32)
*Informal employment*	99 (12.63)	(10.48, 15.15)
*Other*	58 (7.40)	(5.76, 9.46)
**Current health status**		
*EQ-5D Weight 0*.*90 to 1*.*00*	309 (39.41)	(36.04, 42.89)
*EQ-5D Weight 0*.*50 to 0*.*89*	389 (49.62)	(46.12, 53.12)
*EQ-5D Weight <0*.*50*	86 (10.97)	(8.96, 13.36)
**Status of health scheme**		
*Member*	458 (58.42)	(54.92, 61.83)
*Non-member*	326 (41.58)	(38.17, 45.08)
**Social financial safety**		
*Yes*	146 (18.62)	(16.04, 21.51)
*No*	638 (81.38)	(78.49, 83.96)
**Communities**		
*Rural*	458 (58.42)	(54.92, 61.83)
*Urban*	326 (41.58)	(38.17, 45.08)
Income quintile		
* 1st (≤ 5*,*221 BDT*)	195 (24.87)	(21.96, 28.03)
* 2nd (5*,*222–9*,*796 BDT*)	121 (15.43)	(13.07, 18.14)
* 3rd (9*,*797–13*,*445 BDT*)	182 (23.21)	(20.38, 26.31)
* 4th (13*,*446–18*,*137 BDT*)	138 (17.60)	(15.09, 20.43)
* 5th (≥18*,*138 BDT*)	148 (18.88)	(16.28, 21.78)

### Relationship between health scheme and associated determinants

Table 2 shows the result of unadjusted logistic regression analysis using all associated factors with cooperative based health scheme (Co-BHS) membership as the dependent variable. Unadjusted analysis suggested that the Co-BHS membership of female headed household, household size (5 to 6 and more than 8 members), housewife self-reported poor health status score (EQ-5D≤0.490), population those living rural communities, medium (7997–13,445 BDT) and upper medium (13,446–18,137 BDT) income groups of households were the factors found to be significantly associated with enrollment status of health scheme.

**Table 2:** Factors influencing on the determinants of people joining cooperative based health Scheme.

Variables	Model-I^1^		Model-II^2^		
	Unadjusted		Adjusted		
	Odds Ratio (OR)	95% CI^3^ (OR)	Odds ratio (OR)	95% CI^3^ (OR)	VIF^4^
Constant	**-**	**-**	0.37***	(0.07, 2.03)	-
**Sex of household head**					
*Female (Ref)*	1.00	-	1.00	-	-
*Male*	0.14***	(0.09, 0.22)	0.42**	(0.19, 0.95)	1.52
**Age**					
*18–25 (Ref)*	1.00	-	1.00	-	-
*26–35*	0.77	(0.48, 1.24)	0.97	(0.54, 1.74)	3.69
*36–45*	0.95	(0.58, 1.56)	1.36	(0.72, 2.54)	3.2
*46–64*	0.69	(0.42, 1.13)	1.22	(0.63, 2.35)	3.57
*≥65*	1.08	(0.48, 2.43)	1.86	(0.70, 4.96)	1.49
**Marital status**					
*Married (Ref)*	1.00	-	1.00	-	-
*Other*	1.31	(0.83, 2.07)	1.31	(0.73, 2.32)	1.31
**Highest education level**					
*No education*	1.15	(0.49, 2.69)	0.82	(0.29, 2.29)	4.35
*Primary*	1.57	(0.71, 3.43)	1.19	(0.47, 3.03)	2.17
*Secondary*	1.67	(0.76, 3.68)	1.29	(0.52, 3.20)	1.67
*Higher secondary*	1.51	(0.55, 4.12)	1.85	(0.58, 5.85)	1.99
*Higher (Ref)*	1.00	-	1.00	-	-
**Household size**					
*≤ 2 (Ref)*	1.00	-	1.00	-	-
*3–4*	0.95	(0.40, 2.25)	1.37	(0.47, 4.03)	2.57
*5–6*	2.62**	(1.12, 6.12)	4.73***	(1.62, 13.86)	4.15
*7–8*	1.63	(0.65, 4.12)	2.81*	(0.88, 8.93)	3.82
*>8*	8.23***	(1.89, 35.83)	14.63***	(2.78, 77.06)	1.74
**Employment status**					
*Informal employment (Ref)*	1.00	-	1.00	-	
*Worker*	1.49	(0.93–2.38)	1.65*	(0.94, 2.91)	4.43
*Business*	0.90**	(0.54–1.50)	0.80	(0.46, 1.42)	2.83
*House wife*	7.17***	(4.27–12.64)	9.69***	(3.40, 27.55)	3.55
*Farmer*	1.02	(0.53–1.96)	0.89	(0.42, 1.91)	1.86
*Other*	4.09**	(1.99–8.42)	2.99***	(1.37, 6.58)	1.67
**Current health status (EQ-5D Score)**					
*0*.*901≤EQ-5D≤1*.*000 (Ref)*	1.00	-	1.00	-	-
*0*.*500≤EQ-5D≤0*.*900*	1.16	(0.85–1.56)	0.90	(0.62, 1.31)	1.50
*EQ-5D≤0*.*490*	1.67**	(1.01–2.77)	1.27	(0.69, 2.34)	2.60
**Social financial safety**					
*Yes(Ref)*	1.00	-	1.00	-	-
*No*	0.82	(0.57, 1.18)	1.56**	(0.98, 2.47)	4.84
**Communities**					
*Urban (Ref)*	1.00	-	1.00	-	-
*Rural*	1.55***	(1.17–2.07)	0.91	(0.62, 1.32)	2.30
**Income quintile**					
*1st (≤ 5*,*221 BDT*)	0.72	(0.47–1.13)	0.86	(0.49, 1.5)	2.86
*2nd (5*,*222–9*,*796 BDT*)	0.67	(0.41–1.11)	0.80	(0.45, 1.43)	1.98
*3rd (7*,*997–13*,*445 BDT*)	0.66**	(0.42–1.03)	0.69	(0.40, 1.17)	2.46
*4th (13*,*446–18*,*137 BDT*)	0.70**	(0.44–1.14)	0.75	(0.43, 1.3)	2.03
*5th (18*,*138+ BDT*) (*Ref*)	1.00	-	1.00	-	-
*Total observation (N)*			*784*		
^5^*LR chi*^*2*^ *(27)*			*208*.21***		
*Cox-Snell R-square (R*^*2*^*)*			*33*.*30%*		
*Hosmer-Lemeshow’s statistic (Prob > chi*^*2*^*)*			*285*.*38 (0*.*293)*		
*Mean VIF (Max)*			*2*.*67 (4*.*84)*		

^1^Univariate logistic regression model,

^2^Multivariate logistic regression model,

^3^Confident interval,

^4^uncentral variance influential factor,

^5^Likelihood Ratio,

***, ** and * denote 1%, 5% and 10% significance level respectively

### Determinants of health scheme membership

Step up methods were used to enter all factors into one regression model for adjusted analysis (Table 2). This model showed that a number of factors were had greatest influence on the decision to enroll the scheme after controlling for a range of associated covariates. The regression model explains 33.40% of total variation (Cox-Snell R-square R^2^ = 0.334). The diagnostic tests were employed the regression model. Variance inflation factor (VIF) test with its mean (max) value of 2.67 (4.84) indicates that there is no evidence of multicollinearity problem in the regression model [31]. Hosmer and Lemeshow statistic showed no significant difference between the model and observed data (p = 0.293), confirming a good fit of the model to the data [29]. Co-BHS member of male household heads were significantly less likely to enroll this scheme than the female heads (AOR = 0.42, 95% CI: 0.19–0.95, p<0.05). However, household size and occupation were significant factors for enrollment. The result found that, larger households (5 to 6 household members) were significantly more likely to be enrolled in the Co-BHS membership than household with less than three members (AOR = 4.73, 95% CI:1.62–13.86, p<0.001). Based on occupation, housewives were significantly more frequently enrolled in the scheme than the informal employment (AOR = 9.69, 95% CI: 3.40–27.55, p<0.001). Again, those who had coverage under the social financial safety net program were significantly more likely to join the scheme (AOR = 1.56, 95% CI: 0.98–2.47, p<0.05).

## Discussion

In Bangladesh, the informal sector and the number of informal workers have been increasing in recent years but adequate social protection and proper employment benefits including healthcare are sadly deficient for this group [32]. However, a central preoccupation of policy makers is providing appropriate access to health services not only for the workers but also their families as a step on the path to achieving universal health coverage (UHC). Around 30 low- and middle-income countries have introduced health and social health insurance programs, particularly covering the poorer citizens to accelerate UHC [33,34]. However, the goal of universal health coverage becomes more realistic when a larger part of population has access to prepayment and pooling mechanisms [7]. Thus, the cooperative based health scheme discussed in this paper is not only a contribution to improving the coverage of informal sector workers in Bangladesh, but it may also serve as a stepping stone to UHC for Bangladesh.

Health insurance for this specific group of manual workers is warranted in many LMI countries since reliance on out-of-pocket payments for health services leads to a catastrophic burden for many households in such countries and accessibility and utilization of healthcare can be increased for workers and their families at an affordable cost. This study found that a number of factors were significant determinants of health scheme participation including sex of household head, household composition, occupational category, and self-assessed health status of household head and social financial safety. Our result showed that gender of the household head was one of the important predictors of joining this health scheme and female-headed households were more frequently protected under the scheme than male-headed household. We could not find a plausible explanation for this observation but it might be that females have greater awareness of the importance of healthcare than males. We did not find any correlation between the educational level of household head and the enrollment of the scheme although in other settings education has been shown to be an important determinant of having insurance coverage [22]. Our study showed that household size had a significant positive effect on the likelihood of health insurance policy ownership and those who had 5 to 6 members were frequently enrolled in the scheme. It might be that those larger households were more conscious about the financial affordability during illness as affordability is an important barrier to health care [35,36]. A similar association was found regarding the inner occupational categories of informal workers as different occupations had different degrees of participation and different amounts of contribution [37]. Housewives were significantly more frequently enrolled in the scheme than any other workers (Table 2). We believe this is because they directly observe the necessity for healthcare for her own household members during illness than any other groups, however, this specific group might be found more easily for marketing door- to door by sales agents than other groups After controlling for a range of variables, we did not find out any significant relationship with wealth status and enrollment of these occupational group, although socio-economic status groups with lower income are normally less- advantaged [38]. These findings are consistent with the other studies that subsidies make health insurance affordable among the informal workers [20,22]. Again, health status is not only an important determinant of both earnings and capacity for enjoying life but also an important predictor of health insurance coverage [22,38,39]. Univariate analysis showed that that the household head who had relatively lower health status (EQ-5D weight below 0.5) were significantly more likely to join the health scheme than those in better health (0.5 to 1) which indicated the demand for health insurance was higher among individuals who have relatively poor health status. This might be a case of adverse selection related with the voluntary health schemes which jeopardize the economic viability of a health insurance schemes [20]. However, this relation was not observed in multivariate analysis which support the previous findings that health status of the household head was not significant influenced on enrolment [40]. This study has some limitations. One limitation of the current study is that no data were collected on out-of-pocket payments and health care utilization; therefore it was not possible to examine the effect of having health insurance on these two outcomes. Secondly, we did not study insurance-specific attributes, e.g. premiums, co-payments, deductibles, benefits covered and the quality of care in the health facilities where the insured sought care. In spite of some limitations, demographic and personal characteristics were found to be associated with health insurance membership, which can assist the development of intervention programs for informal sector workers to further increase coverage and effect.

## Conclusion

Research on the informal worker’s healthcare and health insurance is of great importance. The study found a number of factors such as female headed households, larger households, occupational category as well as involvement a social financial safety net programs are likely to increase the odds of scheme enrollment. By highlighting which constituencies participate enthusiastically, and which disengage from voluntary insurance programs, findings from this study can be suggestive for policy-makers interested in scaling up health insurance for informal workers in Bangladesh. Shared funding from this large informal sector can generate new resources for healthcare, which is line with the healthcare financing strategy of Bangladesh as well as the recommendation of the World Health Organization for developing social health insurance as part of the path to Universal Health Coverage [7,11].

## Supporting information

S1 Dataset (DTA)
